# Exploring the impact of compound retrieval strategy in the task-switching paradigm

**DOI:** 10.3389/fnhum.2025.1570551

**Published:** 2025-08-05

**Authors:** Fangyuan Zhou

**Affiliations:** ^1^School of Physical Education, Chongqing University of Arts and Sciences, Chongqing, China; ^2^School of Psychology, Shanghai University of Sport, Shanghai, China

**Keywords:** compound retrieval strategy, task-switching paradigm, ERP, N2, switch positivity

## Abstract

**Introduction:**

Task-switching costs are commonly used to measure cognitive control. However, previous research has shown that when participants are not explicitly instructed on task rules, they can adopt the compound retrieval strategy in task-switching paradigms with limited targets, where task-switching costs cannot reflect cognitive control. Nevertheless, it remains unclear whether participants would spontaneously use the compound retrieval strategy when they are explicitly instructed on task rules. This study aimed to investigate this issue.

**Methods:**

In the experiment, 36 participants were recruited to complete two conditions: the four-target condition, in which only four targets were presented and repeated throughout the experiment, and the infinite-target condition, where targets were not repeated.

**Results:**

The results revealed that, compared to the infinite-target condition, task-switching costs were smaller, while response-congruency effects and the N2 difference wave (incongruent trials—congruent trials) were larger in the four-target condition.

**Discussion:**

These findings suggest that participants spontaneously use the compound retrieval strategy in the task-switching paradigm with limited targets.

## 1 Introduction

In task-switching paradigms, participants are required to alternate between different tasks (switch trials) or repeat the same task (repeat trials) in successive trials. Switch trials generally result in slower reaction times (RT) and higher error rates (ER) compared to repeat trials, a phenomenon known as *task-switching costs* (Diamond, 2013; Koch et al., 2018; Luo et al., 2025; Monsell, 2003). Typically, smaller task-switching costs are thought to reflect better cognitive control and cognitive flexibility (Li et al., 2020; Tsai et al., 2017; Zhao et al., 2020).

Due to the limited number of cue and target stimuli generally used in task-switching paradigms, previous research has shown that, in the absence of explicit task-rule instructions, participants can employ a *compound retrieval strategy* to perform the task-switching paradigm (Forrest et al., 2014; Li X. et al., 2019; Logan and Bundesen, 2003, 2004). This strategy involves retrieving a response directly from long-term memory following associative learning of a cue-target compound (e.g., a specific cue combined with a specific target, pressing the “L” key). Since this strategy bypasses cognitive control, task-switching costs are entirely eliminated when participants employ it in the task-switching paradigm (Li X. et al., 2019). However, limited research exists on whether participants spontaneously adopt the compound retrieval strategy when given explicit task-rule instructions. If they do, a reduction in task-switching costs may reflect reliance on this strategy rather than improved cognitive control. Therefore, it is important to explore whether participants spontaneously use the compound retrieval strategy in task-switching paradigms with limited target stimuli, as this could confound interpretations of cognitive control.

We can investigate this issue by manipulating the number of target stimuli. When the number of targets is limited, each cue-target compound repeats in the experiment, forming traces in long-term memory, which allows participants to retrieve the corresponding response for each cue-target compound. However, when targets do not repeat, each cue-target compound appears only once, preventing participants from retrieving the response from long-term memory, thus avoiding the use of the compound retrieval strategy and relying solely on the task rules. To manipulate the number of target stimuli, I adapted the *coordinate switch paradigm* proposed by Schneider (2018). In this paradigm, participants were asked to count hollow dots on a coordinate system. In the vertical task, participants judged whether the top half (quadrants I and II) or the bottom half (quadrants III and IV) contained more dots, while in the horizontal task, they judged whether the left half (quadrants II and III) or the right half (quadrants I and IV) contained more dots. In the infinite-target condition, the quantity and position of dots were randomized with specific constraints (see Method section of Experiment 1), ensuring that each target stimulus was unique across trials. In contrast, the limited-target condition involved a smaller set of fixed target stimuli, allowing for the formation of repeated cue-target associations, thus facilitating the use of compound retrieval strategies. As mentioned earlier, when participants rely solely on the compound retrieval strategy, task-switching costs are completely eliminated. Therefore, I predicted that if participants spontaneously adopted the compound retrieval strategy in the task-switching paradigm with limited targets, task-switching costs in the limited-target condition would be smaller than those in the infinite-target condition.

Apart from task-switching costs, *response-congruency effects* can also be used to assess the strategies participants adopt. In the task-switching paradigm, trials are categorized not only as switch or repeat trials, but also as congruent or incongruent. In congruent trials, the required response is the same across tasks, while in incongruent trials, the response differs. For example, in a numeric task-switching paradigm, according to the task rules (e.g., parity task: odd number = left key, even number = right key; magnitude task: lower than five = left key, higher than five = right key), the numeral “one” is congruent, while the numeral “two” is incongruent. Participants typically show longer RTs and higher ERs in incongruent trials, a phenomenon known as response-congruency effects (Li B. et al., 2019; Schneider, 2015, 2018; Schneider and Logan, 2015; Wendt and Kiesel, 2008).

When participants use the compound retrieval strategy, response-congruency effects arise from direct conflicts between target-response associations (Schneider and Logan, 2015). In contrast, when task rules are applied, these effects are mediated by the rules (Schneider, 2015). Direct conflicts tend to produce larger response-congruency effects than those mediated by task rules (Forrest et al., 2014; Li B. et al., 2019; Meiran and Kessler, 2008; Schneider, 2015). Based on this, I predicted that if participants spontaneously use the compound retrieval strategy in task-switching paradigms with limited targets, response-congruency effects would be larger in the limited-target condition than in the infinite-target condition.

Besides behavioral experiments, numerous event-related potential (ERP) studies have identified neural activities related to task-switching (Czernochowski, 2015; Karayanidis et al., 2011; Karayanidis and Jamadar, 2014). ERPs are voltage fluctuations derived from scalp-recorded electroencephalography (EEG), reflecting phase-locked neural activity associated with sensory, cognitive, or motor processes (Luck, 2014). Specifically, the Switch Positivity and N2 components can be used to assess the strategies participants adopt. When the cue precedes the target, it elicits a positive deflection over parietal-central scalp regions that peaks 300–600 ms after cue onset. This deflection is more pronounced in switch trials than in repeat trials and is known as the Switch Positivity. Additionally, the *Switch Positivity Difference Wave* (SPDW) is derived by subtracting the amplitudes of repeat trials from those of switch trials, which reflects cognitive control (Barceló and Cooper, 2018; Karayanidis and Jamadar, 2014; Kray et al., 2020). When participants use the compound retrieval strategy, cognitive control is not necessary. Therefore, I predicted that if participants spontaneously adopt the compound retrieval strategy in task-switching paradigms with limited targets, the SPDW would be smaller in the limited-target condition than in the infinite-target condition. Moreover, the N2 is a negative-going waveform typically observed over fronto-central scalp regions 200–350 ms after target onset, with its amplitude increasing as conflict strengthens (Folstein and Van Petten, 2007). Thus, the N2 amplitude is larger in incongruent trials than in congruent trials (Chu et al., 2017; Ilan and Polich, 1999; Zhou et al., 2020; Zhou and Qin, 2019). As mentioned earlier, compared to task rules, the compound retrieval strategy involves larger conflict between incongruent and congruent trials. Therefore, I predicted that if participants spontaneously adopt the compound retrieval strategy in task-switching paradigms with limited targets, the N2 difference wave (incongruent trials—congruent trials) would be larger in the limited-target condition than in the infinite-target condition. It should be noted that although other ERP components, such as the P3 component, have also been implicated in decision-making and attention processes, they were not included in the current analysis. The P3 component is typically associated with a broader range of cognitive processes, including stimulus evaluation and categorization (Brydges and Barceló, 2018; Polich, 2007), working memory updating (Ortega et al., 2020), and response selection (Borra and Magosso, 2021), which may not directly align with this study’s specific research questions on task-switching and strategy adoption. Thus, I focused on the SPDW and N2 components, as they are more directly linked to the relevant cognitive control and conflict processes.

In the present study, I aimed to investigate whether participants spontaneously use the compound retrieval strategy in task-switching paradigms with limited target stimuli. According to Shanks (2010), individuals may rely on both cognitive control and associative learning approaches to complete tasks in general scenarios. Therefore, I hypothesized that, in addition to task rules, participants would spontaneously adopt the compound retrieval strategy, as evidenced by: (1) smaller task-switching costs and SPDW (switch—repeat trials) in the limited-target condition compared to the infinite-target condition, and (2) larger response-congruency effects and N2 difference wave (incongruent—congruent trials) in the limited-target condition compared to the infinite-target condition.

## 2 Methods

### 2.1 Participants

The experiment investigated the interaction between trial transition (switch vs. repeat), congruency (congruent vs. incongruent), and condition (four-target vs. infinite-target). A power analysis using G*Power (Faul et al., 2007) indicated that a sample size of 23 participants would be sufficient for detecting a medium effect size (*f* = 0.25), *α* = 0.05, and power = 0.95. Initially, 40 participants were recruited, but 4 were excluded due to excessive EEG noise. Ultimately, data from 36 participants (20 females, mean age = 19.75, SD = 2.52) were analyzed. All participants were right-handed university students, with normal or corrected-to-normal vision, no prior experience with similar cognitive tasks, and no history of neurological or psychiatric disorders. Participants were compensated with 60 RMB (approximately $8.40) for their involvement. This study adhered to the Declaration of Helsinki and was approved by the Ethics Committee of the authors’ university. Additionally, behavioral and EEG data are available on the Open Science Framework.^1^

### 2.2 Procedure

The experiment was conducted over 2 days (Day 1 and Day 2, with a one-week interval between sessions). Participants were randomly assigned to Group 1 or Group 2 (18 participants each). Group 1 completed the four-target condition on Day 1 and the infinite-target condition on Day 2, while Group 2 began with the infinite-target condition on Day 1 and completed the four-target condition on Day 2. While fixed condition orders risk introducing practice effects (Steyvers et al., 2019), fatigue (McMorris et al., 2018), or strategy transfer (Zhao et al., 2020), my counterbalancing design ensures any systematic order effects are equally distributed across conditions (Charness et al., 2012). The 1-week interval further minimizes carryover effects, as procedural learning stabilizes within 48 h (Stickgold and Walker, 2007). Additionally, EEG caps recorded participants’ brain activity during the experiment.

### 2.3 Apparatus, stimuli, and paradigm

The experimental paradigm was programmed with Psychtoolbox software (Brainard, 1997; Kleiner et al., 2007), and stimuli were displayed on a 24-inch monitor.

**Figure 1:**
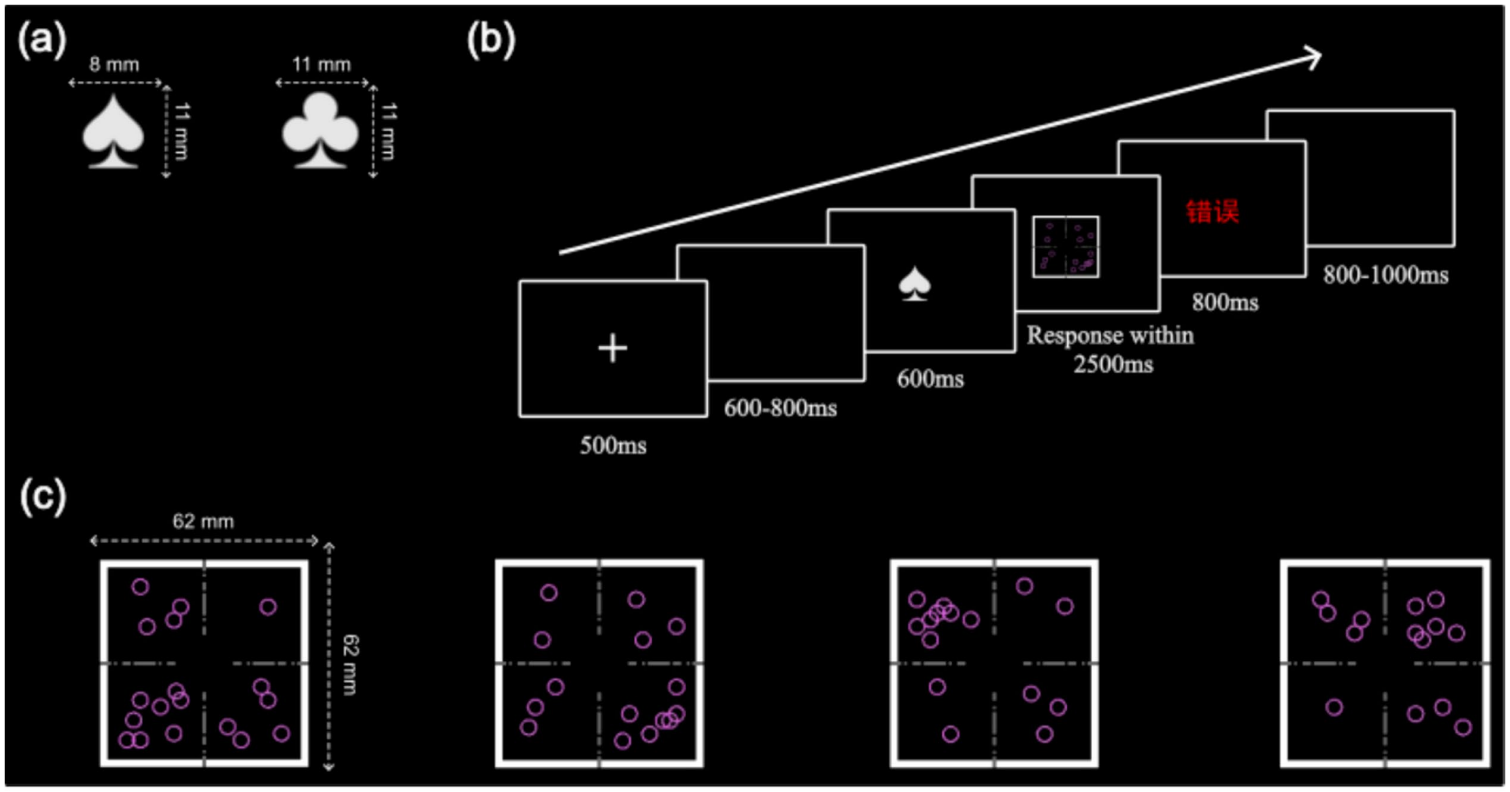
Stimulus materials in the paradigm: **(a)** The two cues in the experiment: “
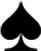
” represents the vertical task, and “
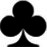
” represents the horizontal task. **(b)** Schematic illustration of a trial timeline. **(c)** The four targets in the four-target condition.

In the infinite-target condition, targets did not repeat during the experiment. Specifically, the positions and quantities of the hollow circles were randomized on each trial, subject to four constraints: (1) the circles could not overlap with the coordinate axes, (2) each quadrant could contain between 1 and 9 circles, (3) the number of circles in the upper halves (quadrants 1 and 2) had to differ from the number in the lower halves (quadrants 3 and 4), and (4) the number of circles in the left halves (quadrants 2 and 3) had to differ from the number in the right halves (quadrants 1 and 4). In the four-target condition, only four distinct targets were presented (see Figure 1c). The order of their presentation was randomized, and no all-repeat trials were allowed (i.e., the cue and target of the current trial were never identical to those of the preceding trial). The selection of four targets was based on the fact that many task-switching paradigms include only four target stimuli (Forrest et al., 2014; Li X. et al., 2019; Manzi et al., 2011).

Each trial began with a 500 ms fixation point, followed by a random blank screen lasting 600–800 ms. A cue then appeared for 600 ms at the center of the screen, with a spade indicating the vertical task and a club indicating the horizontal task (see Figure 1a). After the cue disappeared, the target was presented, and participants had 2,500 ms to respond. If the response was incorrect or exceeded the time limit, feedback was provided for 800 ms; no feedback was given for correct responses. Finally, a blank screen appeared for 800–1,000 ms, randomized across trials (see Figure 1b). Participants first completed a 16-trial practice block and had to achieve at least 80% accuracy to proceed to the formal experiment, which consisted of three 72-trial blocks, totaling 216 trials.

### 2.4 EEG data recording and pre-processing

Participants were tested in a soundproof room while EEG data were recorded using a 64-channel Brain Products system (Brain Products GmbH, Germany; passband: DC-100 Hz; sampling rate: 1000 Hz), with tin electrodes placed on an elastic cap according to the 10–20 system (Böcker et al., 1994). The data were referenced to FCz and re-referenced offline using the average reference. Impedances were kept below 10kΩ throughout recording. Data were processed in MATLAB R2023a with EEGLAB (Delorme and Makeig, 2004), downsampled to 256 Hz, and filtered with a 0.1–30 Hz bandpass filter. Trials were segmented from 300 ms before cue onset to 1,000 ms after target onset. Independent component analysis (ICA) was applied to remove eye artifacts, and baseline correction was performed from −300 to 0 ms before cue onset. Trials with voltage exceeding ±120 μV were excluded. ERP waveforms were averaged across participants and trials, separately for cue-locked and target-locked epochs.

## 3 Data analysis

### 3.1 Behavior

All warm-up, incorrect, and post-incorrect trials were excluded from the RT analysis. Separate factorial ANOVAs for RT and ER were conducted using R version 3.4.2 (Team, 2014). All multiple comparisons were corrected using the Holm-Bonferroni method (Holm, 1979) to control for family-wise error rate.

### 3.2 ERP

**Figure 2:**
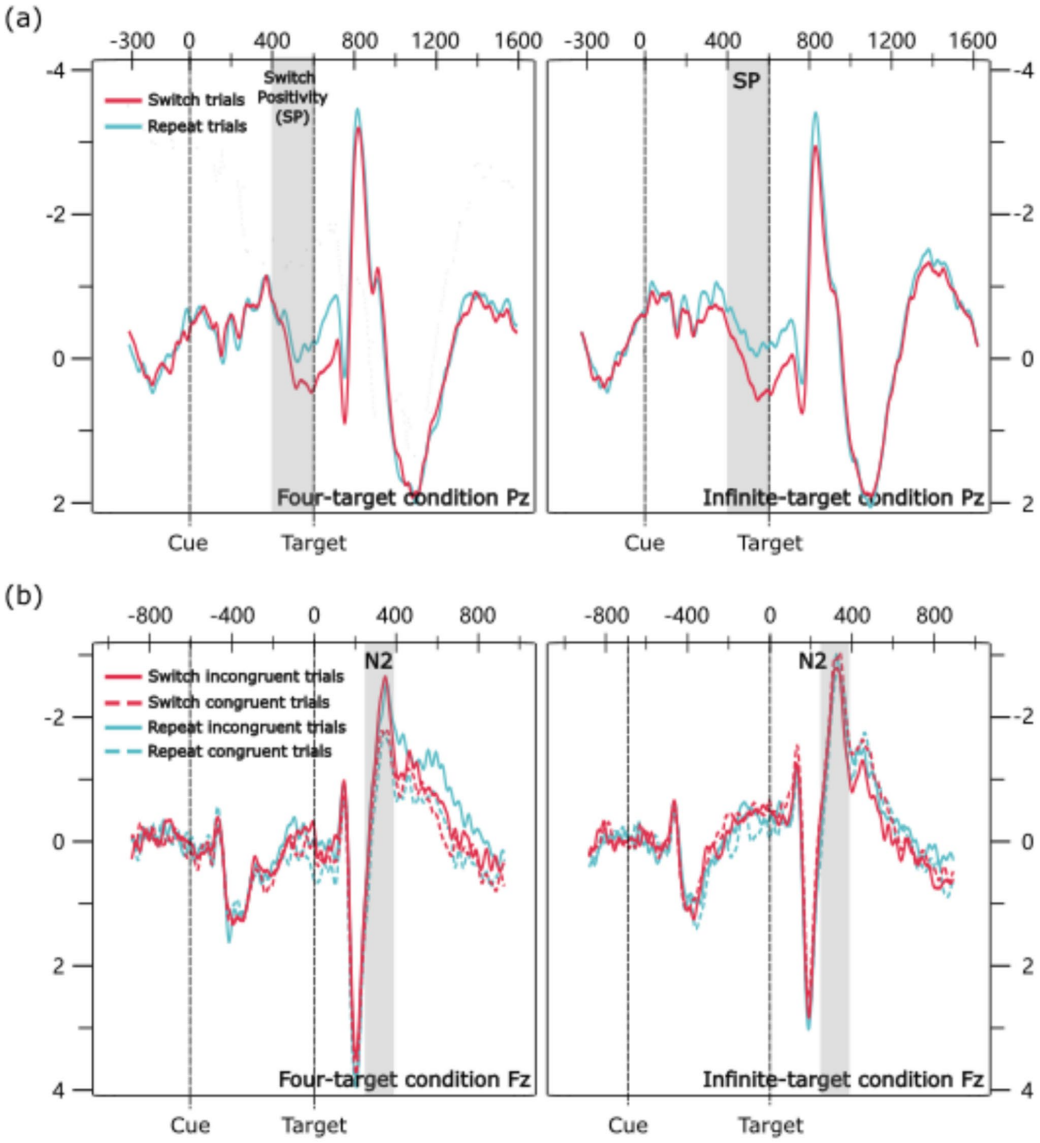
Grand average ERP waveforms: **(a)** Cue-locked ERP waveforms. **(b)** Target-locked ERP waveforms.

All warm-up, incorrect, and post-incorrect trials were excluded from the ERP analysis. Two components were analyzed: Switch Positivity (cue-locked) and N2 (target-locked). Based on previous studies (Chang et al., 2020; Gajewski and Falkenstein, 2011; Zhou et al., 2020; Zhou and Qin, 2019), Switch Positivity was extracted from the Pz channel and N2 from the Fz channel. Based on the waveforms (Figure 2), the time window for Switch Positivity was set to 400–600 ms post-cue, and for N2, 280–380 ms post-target. Mean amplitudes were calculated within ±12 ms of peak amplitude. Two ANOVAs were conducted to examine the amplitudes of Switch Positivity and N2.

## 4 Results

### 4.1 Behavior results

Two separate three-way repeated measures ANOVAs were conducted to examine the effects of three within-subjects factors (trial transition, congruency, condition) on the RT and ER. The results are summarized in Table 1.

**Table 1:** Results of the RT and ER ANOVAs, using trial transition (switch trial, repeat trial), congruency (incongruent trial, congruent trial), and condition (four-target, infinite target) as three within-subjects factors.

Effect	RT				ER			
	*F*	*df*	*p*	*η*^2^_p_	*F*	*df*	*p*	*η*^2^_p_
T	**41.42**	**1, 35**	**<0.001**	**0.54**	**7.55**	**1, 35**	**0.009**	**0.18**
C1	**8.88**	**1, 35**	**0.005**	**0.20**	**105.98**	**1, 35**	**<0.001**	**0.75**
C2	**36.78**	**1, 35**	**<0.001**	**0.51**	**113.36**	**1, 35**	**<0.001**	**0.76**
T × C1	<0.01	1, 35	0.980	<0.01	**20.46**	**1, 35**	**<0.001**	**0.37**
T × C2	**5.47**	**1, 35**	**0.025**	**0.14**	0.91	1, 35	0.347	0.03
C1 × C2	**7.27**	**1, 35**	**0.011**	**0.17**	0.85	1, 35	0.363	0.02
T × C1 × C2	<0.01	1, 35	0.964	<0.01	0.12	1, 35	0.731	<0.01

F, F-statistic; df, degrees of freedom; *η*^2^_p_, partial eta-squared; T, trial transition; C1, congruency; C2, condition; bold represents *p* < 0.05.

### 4.2 RT analysis

#### 4.2.1 RT main effects

The main effect of trial transition was significant; RT for switch trials was longer than for repeat trials (817.72 ms vs. 763.13 ms). The main effect of congruency was significant; RT for incongruent trials was longer than for congruent trials (800.25 ms vs. 780.60 ms). The main effect of condition was significant; RT in the four-target condition was shorter than in the infinite target group (711.93 ms vs. 868.92 ms).

#### 4.2.2 RT task-switching related interactions

**Figure 3:**
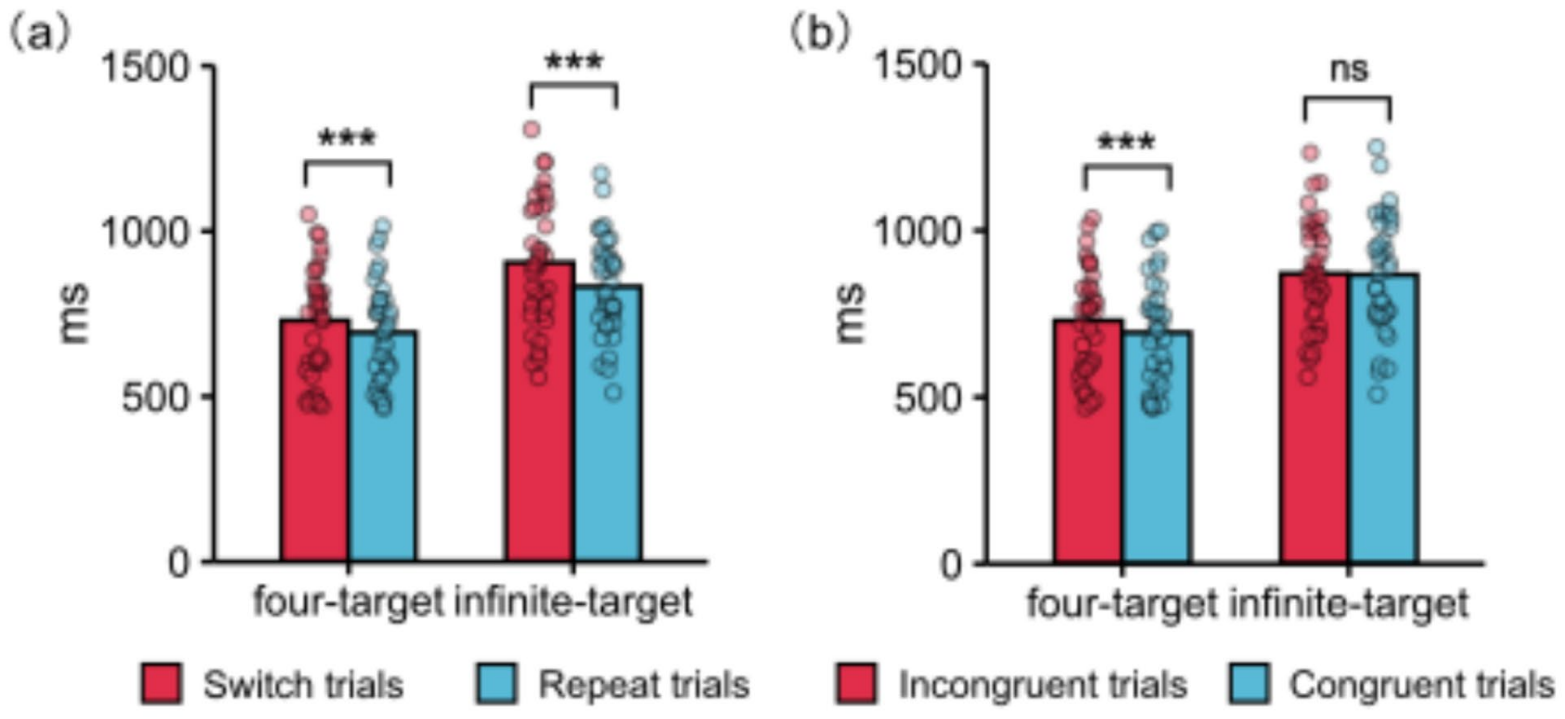
Main behavioral results: **(a)** The interaction between trial transition and condition on RT. Additionally, the RT task-switching costs in the four-target condition were smaller than in the infinite-target condition, *p* = 0.008. **(b)** The interaction between congruency and condition on RT. Additionally, the RT response-congruency effects in the four-target condition were larger than in the infinite-target condition, *p* = 0.005. ***Represents *p* < 0.001 and ns represents *p* > 0.05. Each dot represents data from a participant. ms, milliseconds.

The interaction between trial transition and condition was significant (Figure 3a). Pairwise comparisons showed that in the four-target condition, RT for switch trials was longer than for repeat trials (730.14 ms vs. 693.72 ms), *p* < 0.001, *d* = 0.22. Moreover, in the infinite-target condition, RT for switch trials was longer than for repeat trials (905.30 ms vs. 832.54 ms), *p* < 0.001, *d* = 0.38. However, the task-switching costs in the four-target condition were smaller than in the infinite-target condition (36.42 ms vs. 72.76 ms), *p* = 0.008, *d* = −0.43.

#### 4.2.3 RT congruency-related interactions

The interaction between congruency and condition was significant (Figure 3b). Pairwise comparisons showed that in the four-target condition, RT for incongruent trials was longer than for congruent trials (729.96 ms vs. 693.90 ms), *p* < 0.001, *d* = 0.22. However, in the infinite-target condition, RT between incongruent trials and congruent trials did not show a significant difference (870.54 vs. 867.30), *p* = 0.723. Additionally, the response-congruency effects in the four-target condition were larger than in the infinite-target condition (36.06 vs. 3.24), *p* = 0.005, *d* = 0.46.

### 4.3 ER analysis

#### 4.3.1 ER main effects

The main effect of trial transition was significant; ER for switch trials was higher than for repeat trials (10.07% vs. 8.58%). The main effect of congruency was significant; ER for incongruent trials was higher than for congruent trials (12.70% vs. 5.95%). The main effect of condition was significant; ER in the four-target condition was lower than in the infinite target group (5.33% vs. 13.33%).

#### 4.3.2 ER additional interactions

The interaction between trial transition and congruency was significant. Post-hoc tests showed that in incongruent trials, ER for switch trials was higher than for repeat trials (14.52% vs. 10.88%), *p* < 0.001, *d* = 0.45. However, in congruent trials, ER between switch trials and repeat trials did not show a significant difference (5.62% vs. 6.28%), *p* = 0.169. Additionally, the task-switching costs in incongruent trials were larger than in congruent trials (3.64% vs. -0.66%), *p* < 0.001, *d* = 0.72.

### 4.4 ERP results

Given that participants cannot anticipate congruency before target onset, I analyzed the Switch Positivity amplitude across trial transition and condition. I conducted a two-way repeated measures ANOVA to assess the effects on Switch Positivity. Additionally, I conducted a three-way repeated measures ANOVA to examine the effects of trial transition, congruency, and condition on N2. The results are summarized in Table 2.

**Table 2:** Results of the switch positivity ANOVA (with trial transition and condition as within-subjects factors) and the N2 ANOVA (with trial transition, congruency, and condition as within-subjects factors).

Effect	Switch positivity				N2			
	*F*	*df*	*p*	*η*^2^_p_	*F*	*df*	*p*	*η*^2^_p_
T	**15.17**	**1, 35**	**<0.001**	**0.30**	0.30	1, 35	0.585	0.01
C1	**–**	**–**	**–**	**–**	**5.05**	**1, 35**	**0.031**	**0.13**
C2	0.04	1, 35	0.843	<0.01	**4.20**	**1, 35**	**0.048**	**0.11**
T × C1	–	–	–	–	0.29	1, 35	0.596	0.01
T × C2	0.57	1, 35	0.457	0.02	0.01	1, 35	0.918	<0.01
C1 × C2	**–**	**–**	**–**	**–**	**5.84**	**1, 35**	**0.021**	**0.14**
T × C1 × C2	–	–	–	–	0.56	1, 35	0.460	0.02

F, F-statistic; df, degrees of freedom; *η*^2^_p_, partial eta-squared; T, trial transition; C1, congruency; C2, condition; bold represents *p* < 0.05.

#### 4.4.1 Switch positivity analysis

##### 4.4.1.1 Main effects

The main effect of trial transition was significant; the amplitude for switch trials was larger than for repeat trials (0.53 μV vs. 0.01 μV).

#### 4.4.2 N2 analysis

##### 4.4.2.1 Main effects

The main effect of congruency was significant; the amplitude for incongruent trials was larger than for congruent trials (−2.32 μV vs. -2.73 μV). The main effect of condition was significant; the amplitude in the four-target condition was larger than in the infinite-target condition (−2.10 μV vs. -2.94 μV).

##### 4.4.2.2 Congruency-related interactions

The interaction between congruency and condition was significant. Pairwise comparisons showed that in the four-target condition, the amplitude for incongruent trials was larger than for congruent trials (−1.70 μV vs. -2.50 μV), *p* = 0.003, *d* = 0.26. However, in the infinite-target condition, the amplitude between incongruent trials and congruent trials did not show a significant difference (−2.94 μV vs. -2.95 μV), *p* = 0.983. Additionally, the amplitude of the N2 difference wave (incongruent trials—congruent trials) was larger in the four-target condition than in the infinite-target condition (0.80 μV vs. 0.01 μV), *p* = 0.016, *d* = 0.40.

## 5 Discussion

This study aimed to investigate whether participants spontaneously use the compound retrieval strategy in task-switching paradigms with limited target stimuli. Consistent with my hypothesis, I found that, compared to the infinite-target condition, task-switching costs were smaller, while response-congruency effects and the N2 difference wave were larger in the four-target condition. These findings suggest that participants indeed spontaneously use the compound retrieval strategy.

However, contrary to my hypothesis, the amplitude of SPDW did not show a significant difference between the four-target and infinite-target conditions. Jost et al. (2008) used a dual-cue design, where each task was associated with two cues, and found that not only cognitive control but also cue-switching based on the physical properties of the cue could induce SPDW. Nevertheless, my experiment employed a single-cue design, which is commonly used in many task-switching paradigms (Huang et al., 2022; Luo et al., 2025; Schäfer et al., 2024; Xu et al., 2021; Zhou et al., 2025). In this design, the cue switched whenever a task switch occurred. Therefore, the SPDW in my study may reflect both cognitive control and the switch in the physical properties of the cue. The difference between the four-target and infinite-target conditions was limited to cognitive control, which may not be enough to cause a significant difference in SPDW amplitude between the two conditions. Notably, this null finding could alternatively reflect limited sensitivity to detect small-to-medium effect sizes in my current design (Lakens, 2022). Specifically, the statistical power might have been insufficient to reveal potentially existing differences in SPDW amplitude related to cognitive control demands between conditions. Future studies could adopt a dual-cue design with larger sample sizes to separate the SPDW induced by cognitive control and cue-switching, and explore whether the SPDW induced solely by cognitive control differs between these two conditions.

Based on previous studies (Braver, 2012; Braver et al., 2021; Vandierendonck et al., 2010), task-switching costs are typically attributed to the additional cognitive control required during switch trials. As a result, task-switching costs are often used as a measure of cognitive control. However, in my experiment, I found that when the number of targets was limited (e.g., four targets), participants could spontaneously use the compound retrieval strategy. Consequently, task-switching costs may be influenced not only by cognitive control but also by the compound retrieval strategy. Therefore, task-switching costs in this condition may not fully reflect participants’ cognitive control processes. Moreover, many studies that use task-switching costs as a measure of cognitive control involve a limited number of targets (Feng et al., 2022; Longman et al., 2021; Schäfer et al., 2024; Zhao et al., 2020), raising the question of whether these studies precisely measure cognitive control. This issue warrants further investigation. Additionally, in my experiment, I found that preventing target repetition throughout the experiment effectively restricted participants from using the compound retrieval strategy. Thus, this approach might provide a more reliable way to assess cognitive control. Notably, the smaller task-switching costs in the limited-target condition could stem from enhanced practice effects rather than the compound retrieval strategy, as the restricted target set may facilitate procedural learning (Frings et al., 2024). While my design cannot fully rule out this alternative explanation, this issue warrants further empirical investigation.

The current study has two main limitations. First, only four targets were used to represent the limited-target condition, whereas many task-switching experiments include more than four targets (Graham and Lavric, 2021; Horoufchin et al., 2011; Lui et al., 2025; Yeung, 2025; Yuviler-Gavish and Lazimi, 2025). Future research could include additional target conditions (e.g., four, eight, and sixteen targets) to further explore how different numbers of targets influence participants’ spontaneous use of the compound retrieval strategy. Second, the single-cue design prevents clear separation between SPDW induced by cue-switching and task-switching (cognitive control). Future work should employ the dual-cue design to specifically isolate the cognitive control contribution to SPDW and examine whether this component differs between the infinite-target and limited-target conditions.

## Data Availability

The datasets presented in this study can be found in online repositories. The names of the repository/repositories and accession number(s) can be found in the article/Supplementary material.

## Footnotes

^1^ https://osf.io/j3qs7/?view_only=952fbe79fe1c4dfead869bfd24325675
